# Identification and Fine Mapping of *qCD2*, a Major QTL Governing Leaf Premature Senescence in Rice (*Oryza sativa* L.)

**DOI:** 10.3390/biology15161384

**Published:** 2026-08-13

**Authors:** Bo Yuan, Jiayi Wu, Yang Yang, Keyi Zhang, Jiahe Ren, Jin Liu, Jiayu Wang

**Affiliations:** 1 Key Laboratory of Rice Biology & Genetic Breeding in Northeast China, Ministry of Agriculture and Rural Areas, Rice Research Institute, Shenyang Agricultural University, Shenyang 110866, China; yuanbo@stu.syau.edu.cn (B.Y.); 2024200099@stu.syau.edu.cn (J.W.); 2025200081@stu.syau.edu.cn (Y.Y.); 2024200078@stu.syau.edu.cn (K.Z.); 2024200101@stu.syau.edu.cn (J.R.); 2 Rice Research Institute, Jiangxi Academy of Agricultural Sciences, Nanchang 330200, China

**Keywords:** chlorophyll content, leaf senescence, thermo-sensitivity, fine mapping, rice (*Oryza sativa* L.)

## Abstract

Chlorophyll content is a key determinant of photosynthetic efficiency and grain yield in rice (*Oryza sativa* L.), while premature chlorophyll degradation during the reproductive stage can markedly reduce crop productivity. However, the genetic basis underlying chlorophyll degradation and its environmental responsiveness remains incompletely understood. In this study, an F_2_ population derived from a cross between the japonica cultivar *Shennong0530-9* and the indica cultivar Habataki was used to identify quantitative trait loci (QTLs) associated with chlorophyll content at different developmental stages. A total of 22 QTLs were detected; among these, *qCD2* consistently showed a major and stable effect on chlorophyll degradation after heading. Fine mapping using residual heterozygous lines delimited *qCD2* to a 54.0 kb genomic interval on the short arm of chromosome 2 containing nine predicted genes. Near-isogenic lines (NIL-*qCD2*) carrying the *qCD2* allele exhibited accelerated chlorophyll loss after heading, accompanied by disrupted chloroplast ultrastructure, reduced photosynthetic capacity, and significant decreases in grain yield and grain quality compared with the recurrent parent. Furthermore, the chlorophyll-deficient phenotype became progressively more severe under elevated temperature conditions, indicating that the phenotypic effect associated with *qCD2* is temperature sensitive. Consistent with these physiological changes, the expression patterns of genes involved in chloroplast development, photosynthesis, and leaf senescence were significantly altered in NIL-*qCD2*. Collectively, these results identify *qCD2* as a stable QTL associated with chlorophyll degradation during the reproductive stage and provide a foundation for future identification of the causal gene, providing valuable genetic resources for the molecular breeding of rice with improved photosynthetic efficiency, grain yield, and grain quality.

## Simple Summary

Chlorophyll is essential for photosynthesis, crop growth, yield, and grain quality. However, the genetic basis of chlorophyll degradation and premature leaf senescence in rice remains unclear. In this study, we characterized a rice line showing rapid leaf yellowing at later developmental stages. Genetic analysis identified a major locus on chromosome 2, which was further narrowed to a 54 kb region containing nine predicted genes. Plants carrying the unfavorable allele exhibited accelerated chlorophyll loss, chloroplast damage, reduced photosynthetic capacity, and lower grain yield and quality. The phenotype was aggravated by high temperature. These findings identify an important genomic region involved in maintaining leaf greenness and photosynthetic activity.

## 1. Introduction

Rice (*Oryza sativa* L.) is one of the most important staple food crops worldwide and provides the primary source of calories for more than half of the global population, particularly in Asian countries. With the global population projected to approach 9 billion by 2050, increasing rice productivity has become one of the greatest challenges for ensuring future food security [1,2]. Grain yield is largely determined by photosynthetic efficiency during the reproductive stage, and chlorophyll plays an indispensable role in capturing light energy and driving photosynthesis. Therefore, maintaining chlorophyll content and photosynthetic activity during grain filling is considered an effective strategy for improving biomass accumulation and grain yield in rice [3,4,5]. It has been estimated that approximately 70–80% of the carbon required for grain filling is produced through post-heading photosynthesis, whereas only 20–30% is derived from assimilates accumulated before heading [5]. Consequently, premature chlorophyll degradation and leaf senescence substantially reduce photosynthetic capacity, resulting in significant yield losses and deterioration of grain quality.

Leaf senescence is the final stage of leaf development and represents a highly coordinated developmental process rather than passive tissue degeneration. During senescence, chloroplasts are progressively dismantled, chlorophyll is degraded, photosynthetic proteins are catabolized, and nutrients are remobilized from senescing leaves to developing grains. Appropriate initiation and progression of leaf senescence are therefore essential for balancing photosynthetic productivity and nutrient remobilization, whereas premature senescence frequently results in reduced grain yield [6,7]. Previous studies have demonstrated that leaf senescence is regulated by complex molecular networks involving chloroplast development, chlorophyll metabolism, phytohormone signaling, reactive oxygen species (ROS) homeostasis, autophagy, ubiquitin-mediated protein degradation, and numerous transcription factors [8,9,10,11,12,13,14,15]. In rice, several key regulators, including *OsNAP*, *OsAGO2*, *PLS2*, *PSL50*, *OsCPK12*, and *OsPLS4*, have been shown to participate in chlorophyll degradation, chloroplast maintenance, stress responses, and ROS scavenging [16,17,18,19,20,21]. These findings indicate that leaf senescence is governed by multiple interconnected regulatory pathways rather than by a single genetic mechanism.

Chlorophyll content is a typical quantitative trait controlled by multiple genes and is strongly influenced by environmental conditions. During the past two decades, numerous quantitative trait loci (QTLs) associated with chlorophyll content, stay-green characteristics, and leaf senescence have been identified using diverse rice populations [22,23,24,25,26,27,28,29,30]. These studies have greatly improved our understanding of the genetic architecture of chlorophyll metabolism. Nevertheless, only a limited number of major QTLs have been fine-mapped to sufficiently small genomic intervals for candidate gene identification and functional characterization. Moreover, the genetic basis underlying chlorophyll degradation during the reproductive stage, particularly under elevated temperature conditions, remains incompletely understood. Identification of stable QTLs with clear physiological effects will therefore facilitate the discovery of causal genes and accelerate marker-assisted breeding for improved photosynthetic performance and grain productivity.

In the present study, we identified a major QTL, *qCD2*, associated with chlorophyll degradation using an F_2_ population derived from the cross between the japonica cultivar *Shennong0530-9* and the indica cultivar Habataki. Residual heterozygous lines were subsequently used to fine-map *qCD2* to a 54.0 kb genomic interval. Near-isogenic lines carrying the *qCD2* allele were further characterized to evaluate chlorophyll degradation, chloroplast ultrastructure, photosynthetic performance, grain yield, grain quality, and responses to temperature supply. Our study provides new insights into the genetic basis of chlorophyll degradation during the reproductive stage and establishes a foundation for future identification of the causal gene underlying *qCD2*, thereby facilitating molecular breeding for improved photosynthetic efficiency and grain productivity in rice.

## 2. Materials and Methods

### 2.1. Plant Materials

An early leaf senescence line, designated *Shennong0530-9*, was identified from the backcross progenies derived from the cross between Liaojing454 and Jiangxishimao. The line exhibited premature leaf senescence accompanied by reduced chlorophyll content after heading. To identify the genetic locus responsible for this phenotype, *Shennong0530-9* was crossed with the high-yielding indica cultivar Habataki, which maintains relatively high chlorophyll content during the reproductive stage.

To develop NIL-*qCD2*, *Shennong0530-9* was repeatedly backcrossed with Liaojing454, which served as the recurrent parent, to generate a BC_4_F_2_ population. Genome-wide background analysis was performed using approximately 300 polymorphic SSR markers maintained in our laboratory. A BC_4_F_2_ line carrying the *Shennong0530-9*-derived *qCD2* allele, while exhibiting no detectable polymorphic markers relative to Liaojing454 elsewhere in the genome and retaining the early-senescence phenotype, was selected and designated NIL-*qCD2*.

A total of 210 F_2_ individuals derived from the cross between *Shennong0530-9* and Habataki were used for primary QTL mapping. To minimize the influence of heading date on chlorophyll evaluation, only individuals with a heading-date difference in less than 7 days were included in the analysis; a total of 18 individuals were excluded.

**Figure 1:**
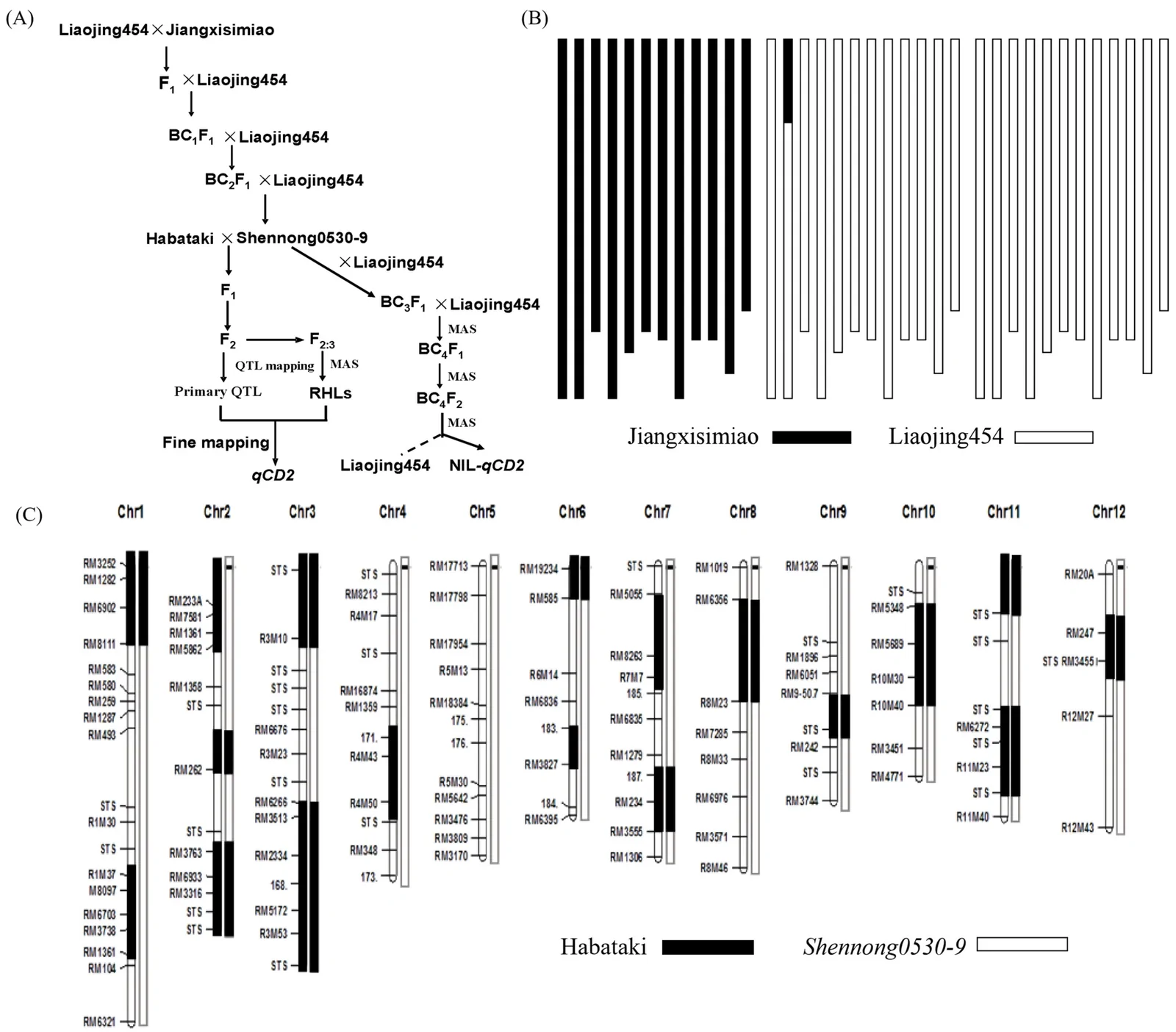
Development of the mapping populations used for fine mapping of *qCD2*. (**A**) Breeding scheme showing the origin of *Shennong0530-9* from the backcross progenies of Liaojing454 *×* Jiangxishimao and the subsequent development of the QTL mapping populations using the cross between *Shennong0530-9* and Habataki. (**B**) Graphical genotypes of the near-isogenic lines (NILs). Solid and open bars represent chromosomal segments derived from Liaojing454 and Jiangxishimao, respectively. (**C**) Graphical genotype of the residual heterozygous line (RHL-*qCD2*) based on 110 SSR markers. Solid and open bars represent chromosomal segments derived from Habataki and *Shennong0530-9*, respectively.

For fine mapping of *qCD2*, five residual heterozygous lines (RHLs) carrying heterozygous chromosomal segments spanning the target region were selected from the F_2_:_3_ population based on genotyping with 110 simple sequence repeat (SSR) markers. Subsequently, a high-resolution mapping population comprising 1264 individuals derived from the RHL-F_2_ and RHL-F_3_ populations was developed for fine mapping. The development of the mapping populations and the graphical genotypes of the selected RHL are shown in Figure 1.

### 2.2. Cultivation and Phenotype Analysis

Field experiments were conducted from 2022 to 2024 at the experimental field of the Rice Research Institute, Shenyang Agricultural University, Shenyang, Liaoning Province, China (123°04′ E, 41°08′ N). The parental lines, F_2_ population, F_2_:_3_ families, NIL-*qCD2*, and the RHL-F_2_ and RHL-F_3_ populations were grown at this site. Seeds were sown between April 18 and 20, and seedlings were transplanted between May 20 and 25. All materials were transplanted as single seedlings, with one seedling per hill, at a spacing of 30 cm between rows and 13.3 cm between plants. The F_2_, RHL-F_2_, and RHL-F_3_ populations were planted and evaluated on an individual-plant basis. For the parental cultivars and NIL-*qCD2*, each plot consisted of 30 plants arranged in three rows, and three independent plots were established for each cultivar. Field management followed standard local agronomic practices.

Leaf chlorophyll status was evaluated using SPAD values measured with a SPAD-502 chlorophyll meter (Konica Minolta, Tokyo, Japan). Measurements were performed at the tillering (65 days after imbibition), Booting (90 days), heading (110 days), and Maturi ty (125 days) stages. For each genotype, SPAD values were measured on the same leaf position of multiple randomly selected plants, and the average value was used for statistical analysis.

The chlorophyll degradation index (CD) was calculated as the ratio of the SPAD value at maturity to that at heading and expressed as a percentage to evaluate the stay-green trait.

### 2.3. Linkage and QTL Analysis

A total of 631 simple sequence repeat (SSR) markers were selected based on the International Rice Genome Sequencing Project database [31] and previous studies reported by Zhang et al. [32]. A genetic linkage map was constructed using MAPMAKER/EXP version 3.0 [33]. Among these markers, 120 polymorphic SSR markers evenly distributed across the rice genome were selected to genotype 210 F_2_ individuals derived from the cross between Shennong0530-9 and Habataki.

SPAD values were used as the phenotypic traits for QTL analysis. QTL mapping was performed using the inclusive composite interval mapping of additive and dominance effects (ICIM-ADD) implemented in QTL IciMapping v4.2 [34]. The walking speed was set to 1.0 cM, and the probability for variables entering the stepwise regression (PIN) was set to 0.001. An LOD score of 2.0 was used as the initial threshold for QTL scanning. Because no permutation test was performed, QTLs detected with relatively low LOD scores were further evaluated according to their reproducibility. QTLs detected only once with LOD scores below 2.4 were excluded from the final QTL set, whereas QTLs repeatedly detected in the F2 and F2:3 populations were retained based on their consistency across the two populations. The phenotypic variance explained (PVE), additive effects, and dominance effects of each retained QTL were estimated. QTL nomenclature followed the recommendations of McCouch et al. [35].

### 2.4. Fine Mapping of qCD2

To fine-map *qCD2*, 110 simple sequence repeat (SSR) markers were used to genotype the F_2_:_3_ population, and five residual heterozygous lines (RHL-*qCD2*) carrying heterozygous segments spanning the target region were identified. These RHLs were subsequently self-pollinated to develop RHL-F_2_ populations. A total of 1264 recessive individuals selected from the RHL-F_2_ and RHL-F_3_ populations were subjected to recombinant analysis for high-resolution mapping of *qCD2*. Recombinant individuals were identified using polymorphic molecular markers, and the *qCD2* locus was further delimited based on the correlation between genotype and phenotype. The polymorphic markers used for fine mapping are listed in Table 1.

### 2.5. Yield and Quality Traits Evaluation

*Shennong0530-9* is a japonica rice cultivar derived from an indica × japonica cross. The NIL-*qCD2* line was developed from Liaojing454 and *Shennong0530-9* through four successive backcrosses. A pair of near-isogenic lines (NIL-*qCD2* and Liaojing454) was selected from the BC_4_F_2_population for agronomic trait evaluation.

Liaojing454 and NIL-*qCD2* were grown in three independent field plots for each genotype. Each plot consisted of 30 plants arranged in three rows, with 10 plants per row. Plants were transplanted with one seedling per hill at a spacing of 30 cm between rows and 13.3 cm between plants. Five plants were sampled from each plot for phenotypic evaluation. The five plants within each plot were considered subsamples, and their mean was used as one plot-level observation. The three independent plots were regarded as three biological replicates.

Major agronomic traits were investigated at maturity, including plant height, effective tiller number, panicle length, grain number per panicle, seed-setting rate, 1000-grain weight, grain length, grain width, grain thickness, and grain quality traits. In addition, SPAD values were measured at the tillering, booting, heading, and maturity stages.

Dried seeds harvested from Liaojing454 and NIL-*qCD2* were stored at room temperature for approximately 3 months to equilibrate seed moisture content prior to milling. Grain quality traits were evaluated according to the Chinese National Standard (NY 147–88), including brown rice rate (BRR), milled rice rate (MRR), chalkiness, and rapid visco analyzer (RVA) characteristics.

### 2.6. Temperature Treatments for NIL-qCD2 and Liaojing454

To investigate the effect of temperature on the chlorophyll-deficient phenotype of NIL-*qCD2*, NIL-*qCD2* and Liaojing454 were grown under natural field conditions with staggered sowing dates. Seeds were sown on 20 April, 1 May, 15 May, 25 May, 5 June, and 15 June, so that plants at different developmental stages could be exposed to the same ambient temperature conditions during overlapping calendar periods. Field air temperature was monitored in situ throughout the experimental period, and the mean temperature corresponding to each developmental stage was calculated for each sowing-date treatment.

Following treatment, the occurrence and severity of leaf yellowing were evaluated, and chlorophyll content was estimated by measuring SPAD values.

### 2.7. Transmission Electron Microscopy (TEM) Analysis

Chloroplast ultrastructure was examined using leaves collected from Liaojing454 and NIL-*qCD2* at the heading stage. Green and senescent leaf tissues from NIL-*qCD2*, together with the corresponding leaf tissues from Liaojing454, were excised and immediately fixed in 2.5% glutaraldehyde and 1% osmium tetroxide prepared in 0.2 mol L^−1^ phosphate buffer (pH 7.2) at 4 °C.

After fixation for 5 h, the samples were dehydrated through a graded ethanol series (50%, 70%, 80%, 95%, and 100%) followed by acetone, embedded in epoxy resin, sectioned into ultrathin slices, and observed using a Hitachi HT7650 transmission electron microscope (Hitachi, Tokyo, Japan).

### 2.8. Photosynthetic and Chlorophyll Fluorescence Kinetic Parameters

At the heading stage (110 days after imbibition), the net photosynthetic rate (Pn), stomatal conductance (Gs), intercellular CO_2_ concentration (Ci), transpiration rate (Tr), and chlorophyll fluorescence parameters were measured on the flag leaves. Gas exchange parameters (Pn, Gs, Ci, and Tr), together with non-photochemical quenching (qN) and electron transport rate (ETR), were determined using a LI-6400 Portable Photosynthesis System equipped with a 6400-40 Leaf Chamber Fluorometer (LI-COR Biosciences, Lincoln, NE, USA). Initial fluorescence (Fo) and the maximum quantum efficiency of PSII (Fv/Fm) were measured using a Handy PEA chlorophyll fluorometer (Hansatech Instruments, King’s Lynn, UK) after 20 min of dark adaptation. All measurements were conducted between 08:30 and 11:00 h on sunny days at approximately 25 °C, following the manufacturers’ instructions. Three biological replicates were analyzed for each genotype.

### 2.9. RNA Extraction and Expression Analysis

Leaf samples were collected from NIL-*qCD2* and Liaojing454 at the onset of premature senescence. The uppermost fully expanded leaves on the main culms were sampled, with tissues collected from the same position within the distal one-third of each leaf. Samples were collected between 9:00 and 10:00 a.m., immediately frozen in liquid nitrogen, and stored at −80 °C. Each biological replicate consisted of pooled leaf tissues from three to five independent plants, with three biological replicates. Total RNA was extracted using the RNAprep Pure Plant Kit (Tiangen Biotech, Beijing, China) according to the manufacturer’s instructions.

Approximately 2.0 μg of total RNA from each sample was reverse-transcribed into first-strand cDNA using the HiScript III 1st Strand cDNA Synthesis Kit (+gDNA wiper) (Vazyme, Nanjing, China) according to the manufacturer’s instructions. Quantitative real-time PCR (RT-qPCR) was performed using the ChamQ SYBR Color qPCR Master Mix (Vazyme, Nanjing, China) on a 7500 Real-Time PCR System (Applied Biosystems, Foster City, CA, USA). According to the manufacturer’s instructions. *ACTIN* was used as the internal reference gene, and the relative gene expression levels were calculated using the 2^−ΔΔCt^ method. Each sample was analyzed with three biological replicates.

Some of the gene-specific primers used for *psaA*, *psbA*, *rbcL*, *V1*, *RpoTp*, *HEMA1*, *PORA*, *CAO*, *OsGK*, *cab1R*, *cab2R*, *RpoA, rbcS and Actin* were adopted from Dong et al. [36] and Wu et al. [37], whereas the remaining primers were designed for this study. All primer sequences are listed in Table S1.

### 2.10. Statistical Analysis

Statistical analyses were performed using GraphPad Prism version 7.0 (GraphPad Software, San Diego, CA, USA) and IBM SPSS Statistics version 25.0 (IBM Corp., Armonk, NY, USA). Data are presented as the mean ± standard deviation (SD) unless otherwise indicated. For field experiments, the mean value of the sampled plants within each plot was treated as one biological replicate. Differences between Liaojing454 and NIL-*qCD2* were evaluated using Student’s *t*-test in GraphPad Prism. Comparisons among multiple groups were performed using one-way analysis of variance (ANOVA), followed by Duncan’s multiple range test in SPSS. Differences were considered statistically significant at *p* < 0.05 and highly significant at *p* < 0.01.

## 3. Results

### 3.1. Detection of QTLs for Chlorophyll Content Traits

**Figure 2:**
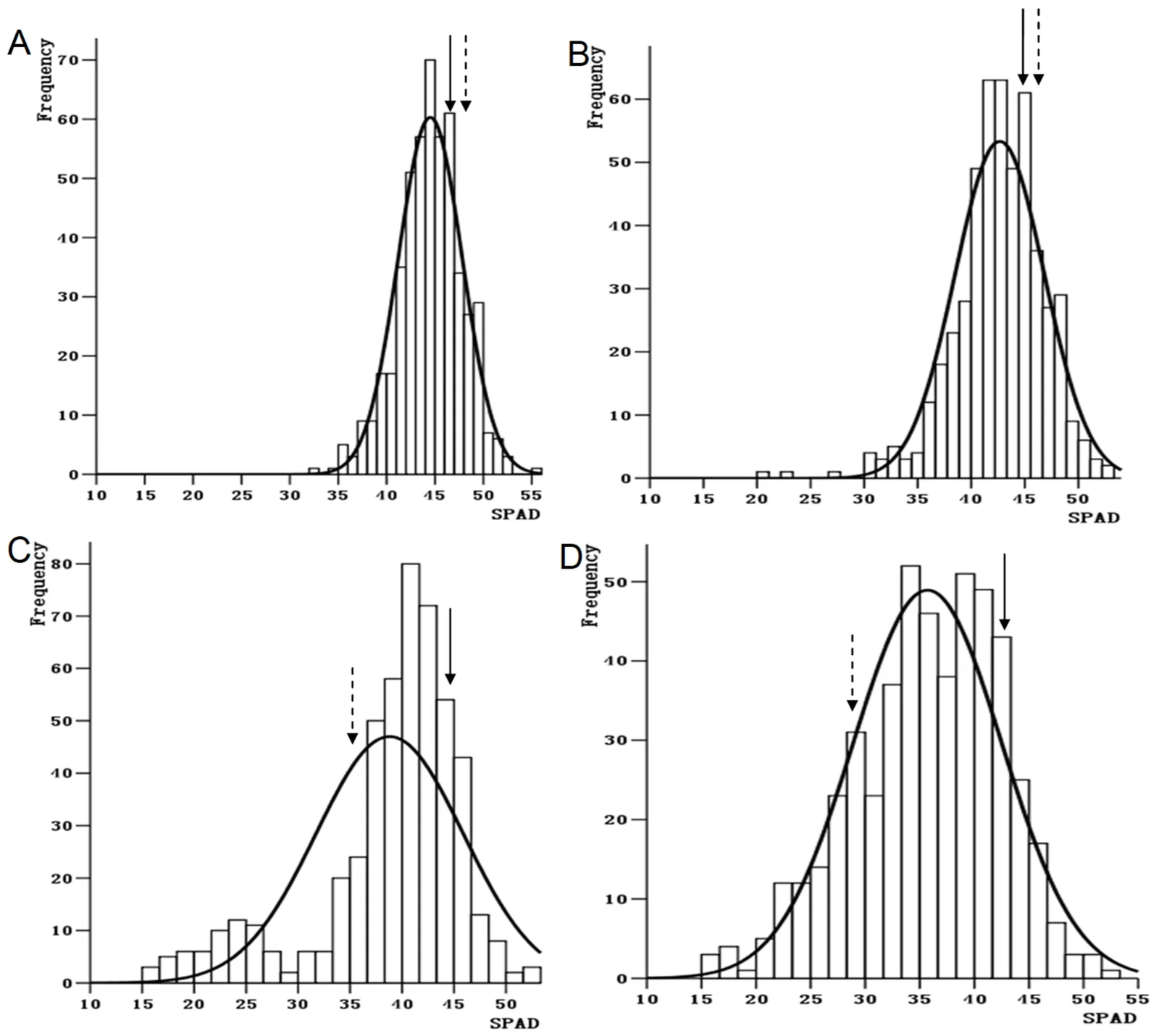
The SPAD value distribution of F2 population plants from the cross of *Shennong0530-9* × Habataki at different stages. (**A**) SPAD value of flag leaves at the late-tiller stage; (**B**) SPAD value of flag leaves at booting stage; (**C**) SPAD value of flag leaves at heading stage; (**D**) SPAD value of flag leaves at maturity stage. (↓and ⇣ represent Habataki and *Shennong0530-9* chlorophyll content, respectively; T, Tillering; B, Booting; H, Heading; M, Maturity, 65, 90, 110 and 125 days after imbibition, respectively.).

Significant differences in chlorophyll content were observed between *Shennong0530-9* and Habataki after the heading stage. Compared with Habataki, *Shennong0530-9* exhibited a much more rapid decline in chlorophyll content during the late developmental stages. In the F_2_ population derived from the cross between these two parents, SPAD values showed continuous variation at the tillering, booting, heading, and maturity stages, indicating that chlorophyll content is a typical quantitative trait suitable for QTL analysis (Figure 2; Table 2).

**Figure 3:**
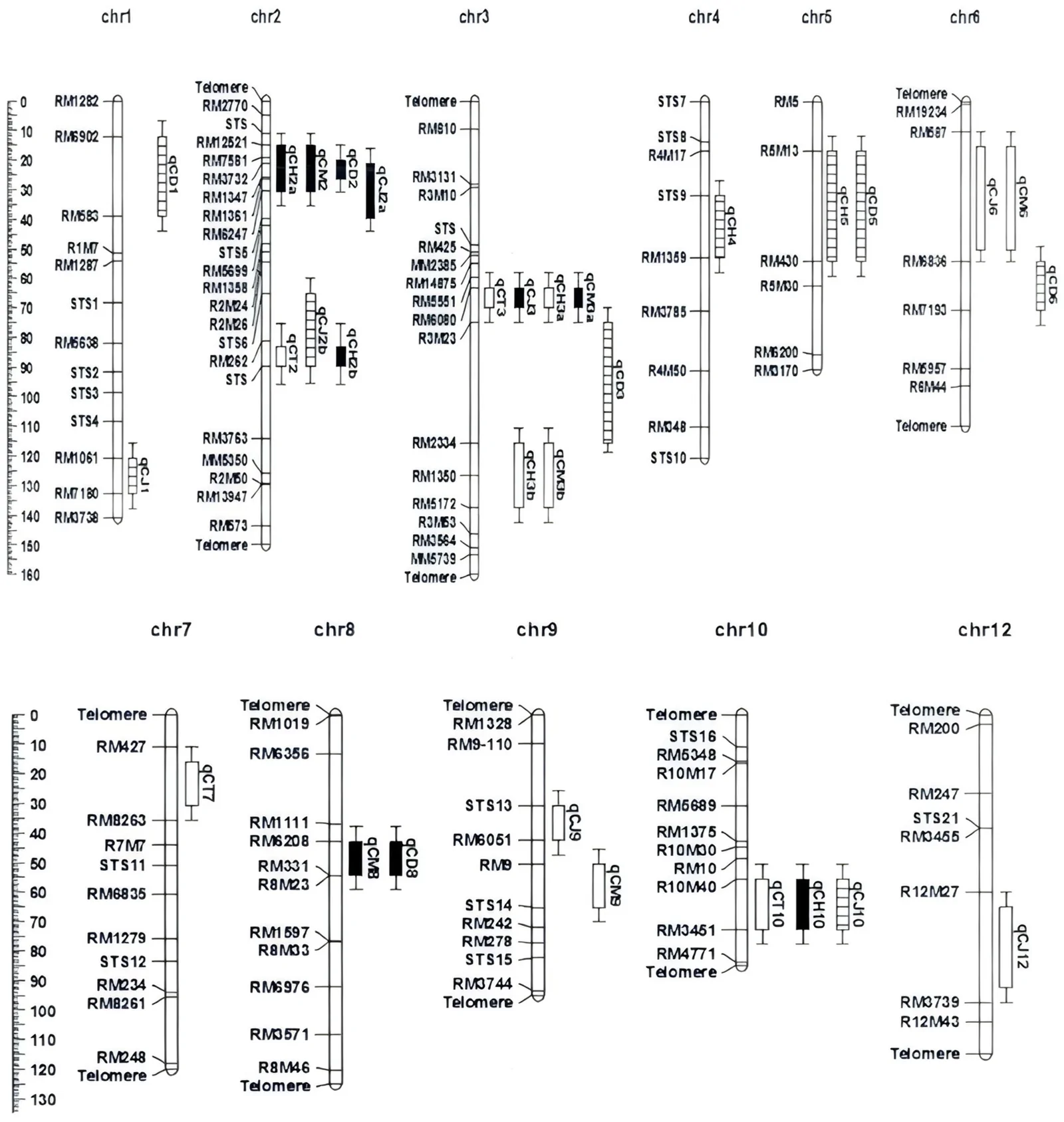
Genetic linkage map showing for chlorophyll content putative QTL positions detected in the F_2_ and F_2_:_3_ populations at different stages (Open boxes represent the QTL mapping intervals detected in the F_2_ population, hatched boxes represent the QTL mapping intervals detected in the F_2_:_3_ population, and solid black boxes represent the co-localized QTL intervals detected in both populations).

QTL mapping in the F_2_ population identified a total of 22 QTLs associated with chlorophyll content and chlorophyll degradation on chromosomes 2, 3, 6, 7, 8, 9, 10, and 12 (Figure 3; Table 3). These QTLs individually explained 4.51–24.65% of the phenotypic variation. Among them, four QTLs were detected at the tillering stage, five at the booting stage, five at the heading stage, six at the maturity stage, and two were specifically associated with chlorophyll degradation, accounting for 36.73%, 49.77%, 50.82%, 59.18%, and 22.05% of the total phenotypic variation, respectively.

Several QTLs detected at different developmental stages were co-localized, suggesting that they may represent pleiotropic loci controlling chlorophyll accumulation throughout plant development. In particular, a major QTL located on the short arm of chromosome 2, designated *qCD2* (corresponding to *qCH2a*, *qCM2* and *qCJ2a* at different developmental stages), was consistently detected between markers STS5 and RM1347. The *Shennong0530-9* allele at this locus decreased SPAD values and was associated with the premature leaf senescence phenotype. *qCD2* was regarded as a stable major QTL because it was repeatedly detected in both the F_2_ and F_2_:_3_ populations and co-localized with SPAD-related QTLs across multiple developmental stages, despite variation in its estimated PVE between populations. Therefore, *qCD2* was selected for subsequent fine-mapping analyses.

### 3.2. Fine Mapping of qCD2

**Figure 4:**
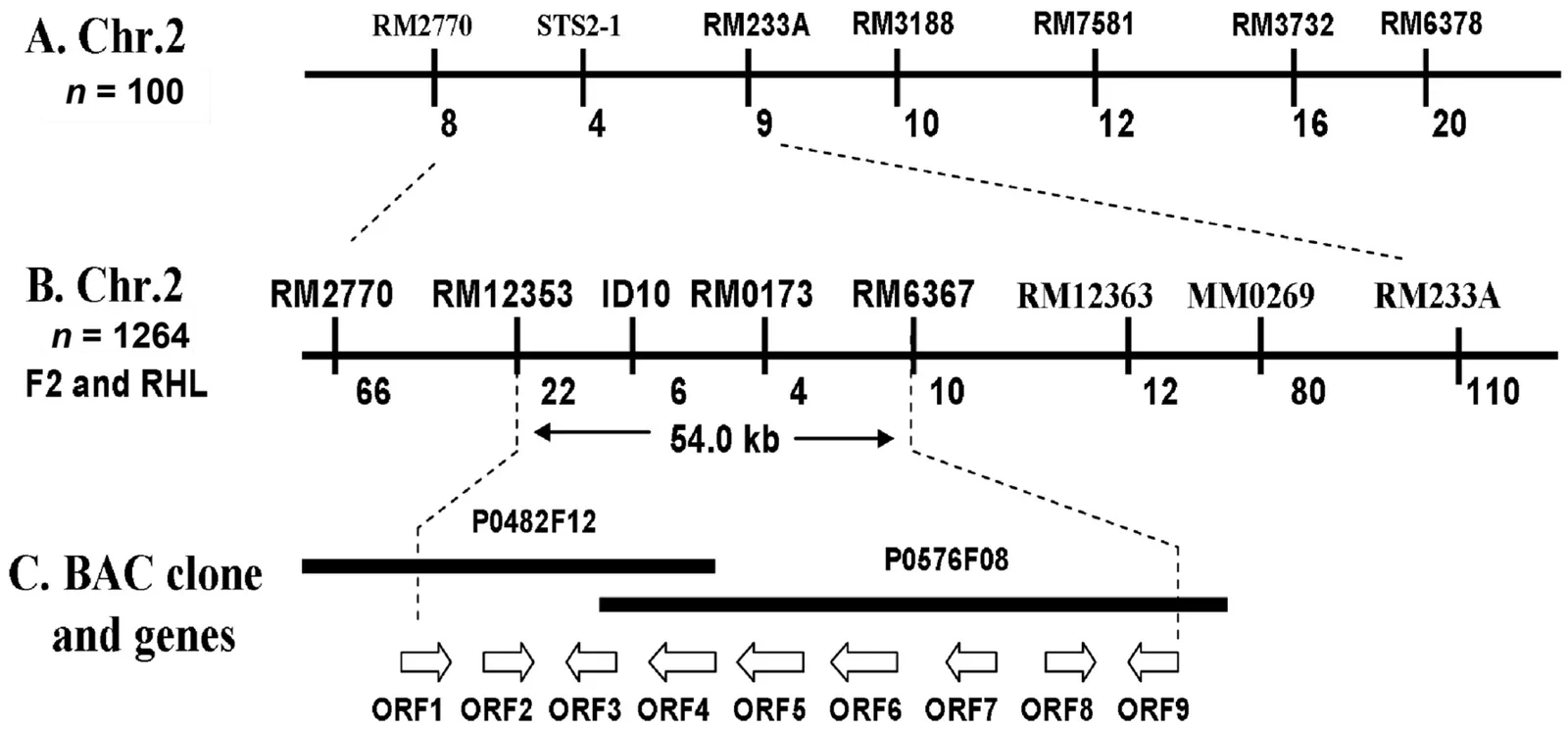
Fine-mapping of the *qCD2* on Chromosome 2 in rice. (**A**,**B**) *qCD2* was fine mapped in a region between markers RM2770 and RM233A; (**C**) Physical map of the *qCD2* region showing the two BAC clones (P0482F12 and P0576F08) and the predicted genes (ORF1–ORF9). The horizontal black bars indicate the BAC clones, and the arrows indicate the predicted genes.

To further delimit the *qCD2* locus, bulked segregant analysis (BSA) was performed using leaf-senescent individuals from the F_2_:_3_ and RHL-*qCD2* populations [38,39,40]. A total of 100 early-heading F_2_ individuals derived from the cross between *Shennong0530-9* and Habataki that exhibited the typical premature leaf senescence phenotype were selected for preliminary linkage analysis. The *qCD2* locus was initially mapped to the interval between the SSR markers RM2770 and RM233A (Figure 4).

Based on the preliminary mapping results, a fine-mapping population consisting of 1264 individuals derived from the F_2_:_3_ and RHL-F_2_ populations was subsequently developed. Eight polymorphic SSR markers located within the target region were screened and selected for recombinant screening. Linkage analysis further narrowed *qCD2* to a 54.0 kb genomic interval flanked by markers RM12353 and RM6367 on the short arm of chromosome 2 (Figure 4).

According to the Rice Annotation Project Database (RAP-DB), the 54.0 kb interval contains nine predicted open reading frames (ORFs) (Table 4). These genes represent the primary candidates for subsequent functional characterization and identification of the causal gene underlying *qCD2*.

### 3.3. Yield-Related Traits and Grain Quality in Near-Isogenic Line NIL-qCD2

Compared with Liaojing454, the biological yield and grain yield of NIL-*qCD2* were significantly reduced. In addition, the number of effective tillers per plant, grain number per panicle, seed-setting rate, and thousand-grain weight decreased from 13.87 to 12.75, from 172.03 to 161.43, from 95.62% to 93.25%, and from 24.41 g to 23.25 g, respectively (Table 5).

Grain quality was also significantly impaired in NIL-*qCD2* (Table 5). Compared with Liaojing454, the chalky grain rate and chalkiness increased from 15.05% to 19.01% and from 8.30% to 14.20%, respectively, whereas the milled rice rate decreased from 74.11% to 71.51%.

Collectively, these results indicate that the *qCD2* locus is associated with variation in multiple yield- and grain quality-related traits during the reproductive stage.

### 3.4. Measurement of the Chlorophyll Content and Chloroplasts

**Figure 5:**
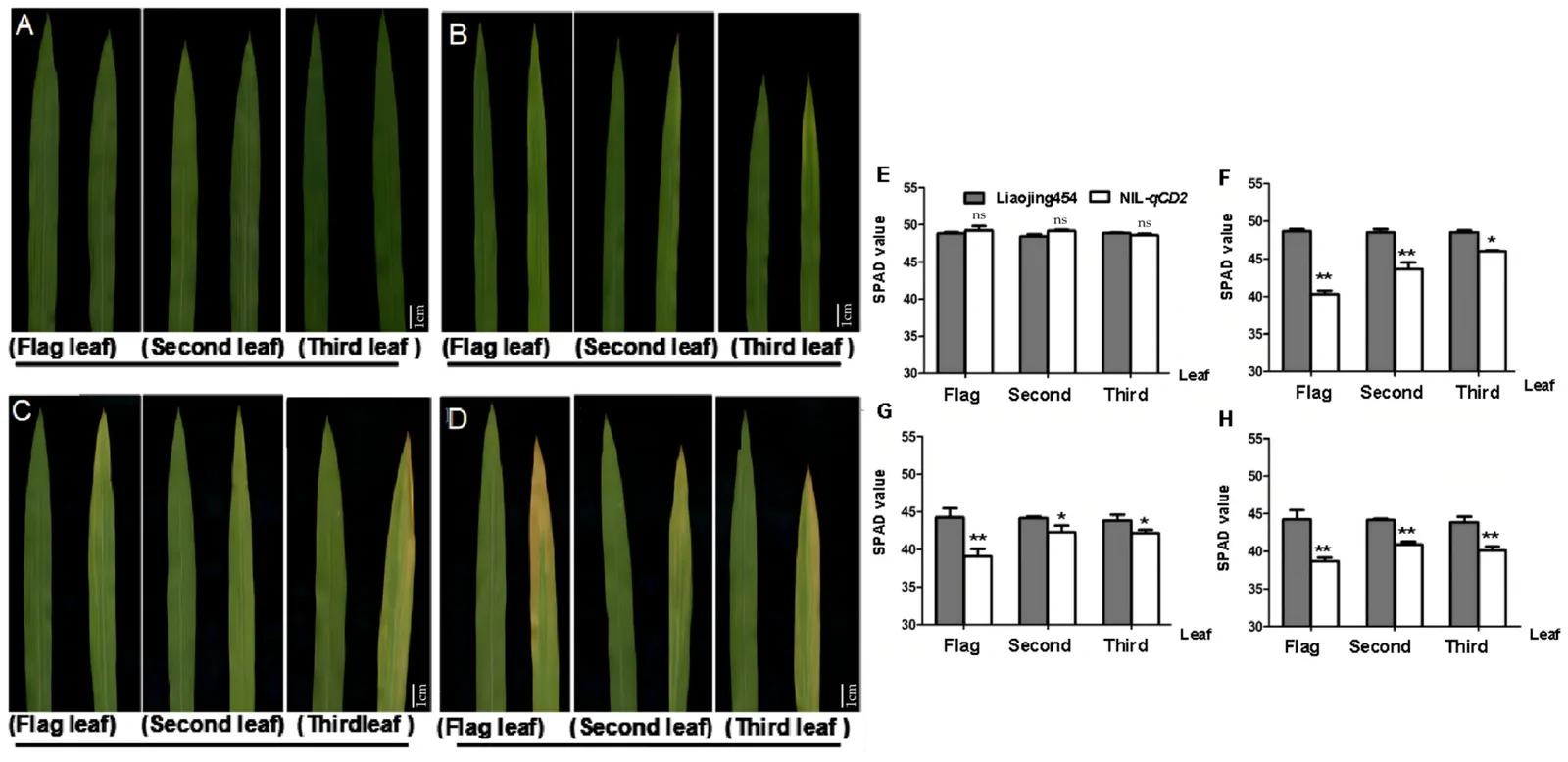
Comparison of leaf morphology, SPAD values, and chloroplast ultrastructure between Liaojing454 and NIL-*qCD2*. (**A**–**D**) Morphological characteristics of the flag, second, and third leaves in Liaojing454 and NIL-*qCD2* at the tillering (**A**), booting (**B**), heading (**C**), and maturity (**D**) stages. For each leaf position, Liaojing454 is shown on the left and NIL-*qCD2* on the right. (**E**–**H**) SPAD values of the flag, second, and third leaves at the tillering (**E**), booting (**F**), heading (**G**), and maturity (**H**) stages. Data are presented as the mean ± SD of three biological replicates. * and ** indicate significant differences at *p* < 0.05 and *p* < 0.01, respectively, according to Student’s *t*-test; ns, not significant.

Relative chlorophyll content was evaluated using SPAD measurements at different developmental stages. No significant difference in chlorophyll content was observed between the two genotypes at the tillering stage. However, from the booting stage onward, chlorophyll content in the flag, second, and third leaves of NIL-*qCD2* was significantly lower than that of Liaojing454. The difference became progressively greater after heading, indicating an earlier and more pronounced decline in SPAD values in NIL-*qCD2* during the reproductive stage (Figure 5A–H).

**Figure 6:**
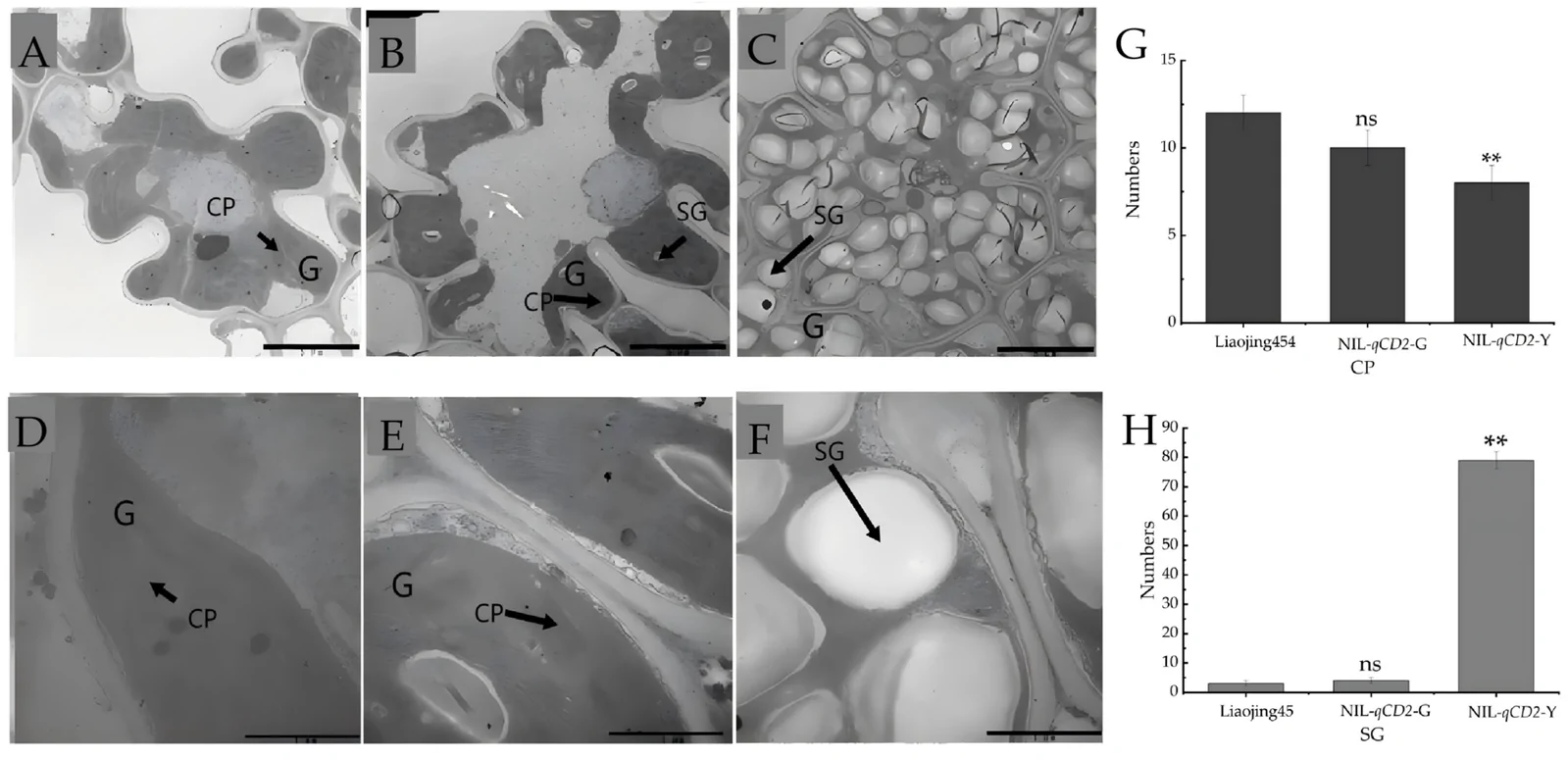
Transmission electron microscopy (TEM) images of chloroplast ultrastructure in flag leaves of Liaojing454 and NIL-*qCD2* at the heading stage. (**A**–**C**) Low-magnification images (8000×; scale bars = 5 μm). (**D**–**F**) High-magnification images (40,000×; scale bars = 1 μm). (**A**,**D**) Liaojing454; (**B**,**E**) green leaf tissues of NIL-*qCD2*; (**C**,**F**) senescent leaf tissues of NIL-*qCD2*; (cp, chloroplast; G, grana; SG, starch grain); Quantification of chloroplast number (**G**) and starch grain number (**H**). The numbers of chloroplasts and starch grains were counted within an area of 4.0 × 10^−6^ cm^2^. Values are presented as the mean ± SD. Each material was compared with Liaojing454 using Student’s *t*-test. **, *p* < 0.01; ns, not significant.

To further characterize the cellular phenotype associated with chlorophyll loss, chloroplast ultrastructure was examined by transmission electron microscopy (TEM) at the heading stage (Figure 6A–F). No obvious differences in chloroplast morphology were observed between Liaojing454 and the green leaves of NIL-*qCD2* (Figure 6A,B,D,E). In contrast, chloroplasts in the senescent leaf tissues of NIL-*qCD2* exhibited severe structural disruption, including degradation of chloroplast membranes and a marked reduction in chloroplast number (Figure 6). Moreover, starch grains accumulated extensively in the remaining chloroplasts of NIL-*qCD2* compared with Liaojing454 (Figure 6C,F). These observations demonstrate that accelerated chlorophyll loss in NIL-*qCD2* was accompanied by pronounced alterations in chloroplast ultrastructure.

### 3.5. Photosynthetic and Chlorophyll Fluorescence Parameters

Photosynthetic gas-exchange parameters were measured to evaluate the effects of *qCD2* on photosynthetic performance (Table 6). Compared with Liaojing454, the yellow leaves of NIL-*qCD2* exhibited a 30.0% reduction in net photosynthetic rate (Pn). Stomatal conductance (Gs) and transpiration rate (Tr) also decreased significantly, whereas intercellular CO_2_ concentration (Ci) showed no significant difference between the two genotypes. In contrast, the green leaves of NIL-*qCD2* exhibited significantly lower stomatal conductance (Gs) and transpiration rate (Tr) than those of Liaojing454, whereas the net photosynthetic rate and the other measured photosynthetic parameters did not differ significantly between the two genotypes. These results indicate that the reduced Gs and Tr did not substantially affect photosynthetic carbon assimilation before visible leaf senescence.

Chlorophyll fluorescence parameters were further analyzed to evaluate PSII photochemical activity (Table 6). The maximum quantum efficiency of PSII (Fv/Fm) showed only a slight decrease from 0.84 in Liaojing454 to 0.83 in the yellow leaves of NIL-*qCD2*. However, the initial fluorescence (F_0_) increased from 177.67 to 194.22. In addition, non-photochemical quenching (qN) and electron transport rate (ETR) decreased by approximately 26% and 37%, respectively, in the yellow leaves of NIL-*qCD2*. These results indicate reduced photosynthetic performance and impaired PSII activity in the senescent leaves of NIL-*qCD2*.

### 3.6. Temperature Response for NIL-qCD2 and Liaojing454

**Figure 7:**
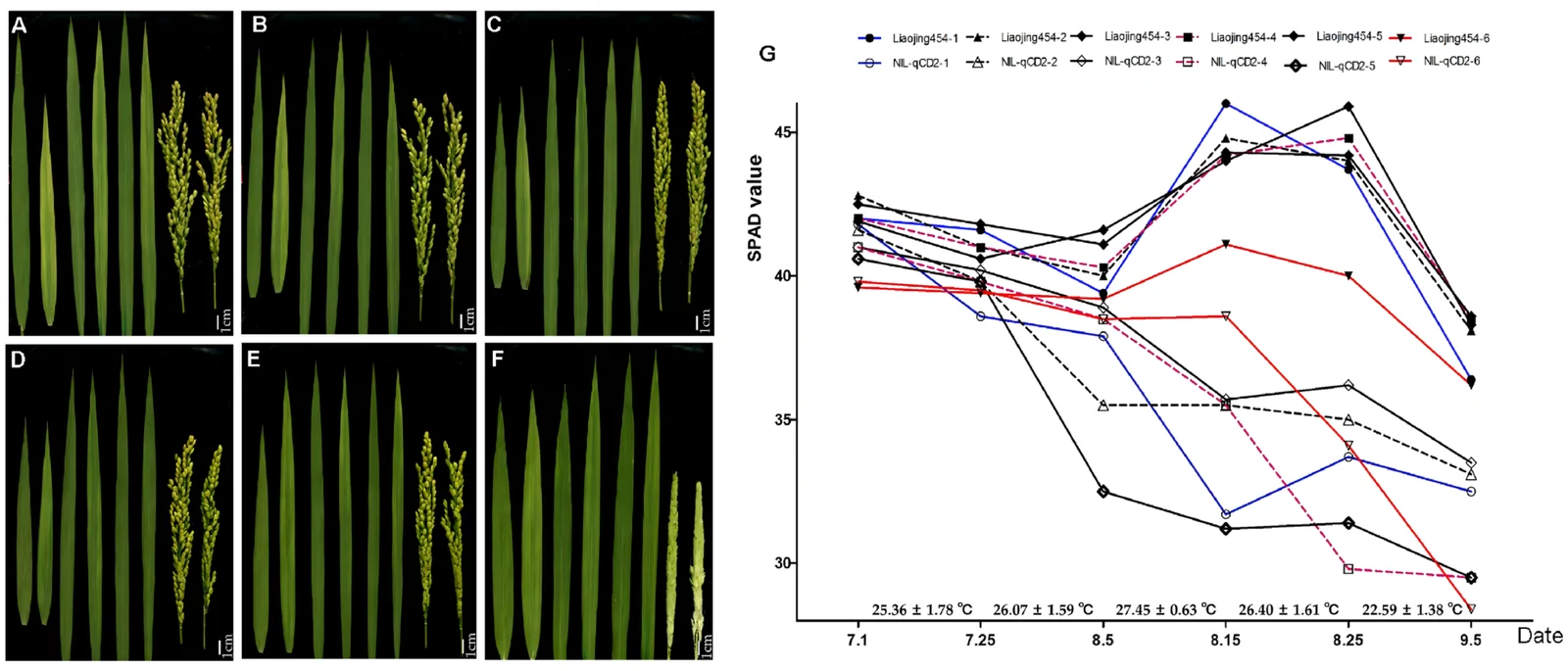
Temperature response for NIL-*qCD2* and Liaojing454. (**A**–**F**) Premature leaf senescence phenotypes of Liaojing454 and NIL-*qCD2* at the late growth stage under six sowing dates: 20 April (**A**), 1 May(**B**), 15 May (**C**), 25 May (**D**), 5 June (**E**), and 15 June (**F**). (**G**) Changes in SPAD values of Liaojing454 and NIL-*qCD2* under different sowing dates. The temperatures shown in the figure represent the mean values of daily average temperatures between the dates indicated on the left and right sides, with error bars calculated from the corresponding daily records. The data were derived from field observations.

The results indicated that NIL-*qCD2* was sensitive to elevated temperatures and that its premature leaf senescence was primarily regulated by temperature rather than developmental stage. From the booting stage onward, increasing ambient temperatures were accompanied by a gradual transition of the leaves from green to a senescent phenotype, together with a marked decline in chlorophyll content (Figure 7A–F). When the daily mean temperature consistently reached the range of 26–28 °C, NIL-*qCD2* leaves began to exhibit pronounced and irreversible premature senescence, suggesting that this temperature range may represent an approximate threshold for the transition from a normal green-leaf phenotype to premature senescence (Figure 7G). Collectively, these results demonstrate that elevated temperatures promote chlorophyll degradation and induce premature leaf senescence in NIL-*qCD2*, and that the development of this phenotype is predominantly associated with temperature rather than the developmental stage itself.

### 3.7. Transcript Expression Levels of Photosynthesis-Related Genes

**Figure 8:**
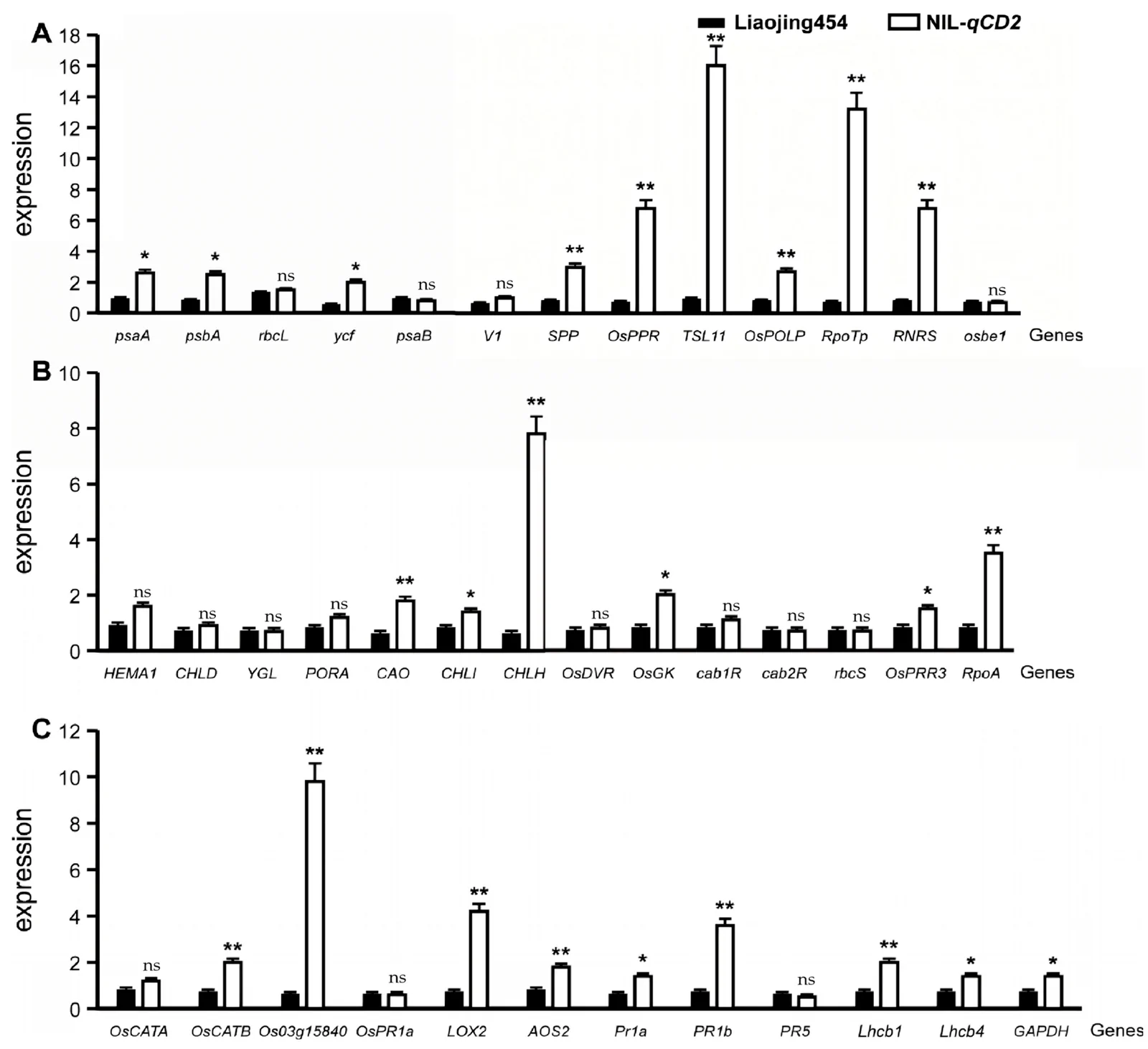
Relative expression levels of genes associated with chloroplast development, chlorophyll biosynthesis, photosynthesis, and leaf senescence in Liaojing454 and NIL-*qCD2* determined by quantitative real-time PCR (RT-qPCR). (**A**) Genes related to chloroplast development. (**B**) Genes related to chlorophyll biosynthesis and photosynthesis. (**C**) Genes related to leaf senescence. Gene expression levels in Liaojing454 were normalized to 1.0, and relative expression levels in NIL-*qCD2* were calculated accordingly. Data are presented as the mean ± SD of three biological replicates. * and ** indicate significant differences at *p* < 0.05 and *p* < 0.01, respectively, according to Student’s *t*-test; ns, not significant.

To investigate the molecular basis underlying the chloroplast defects and reduced photosynthetic performance observed in NIL-*qCD2*, the transcript levels of genes involved in chloroplast development, chlorophyll biosynthesis, photosynthesis, and leaf senescence were analyzed by quantitative RT-PCR (Figure 8).

Genes associated with chloroplast development exhibited significant transcriptional alterations in NIL-*qCD2* (Figure 8A). Among these, the transcript levels of *SPP*, *OsPPR*, *TSL11*, *OsPOLP*, *RpoTp*, and *RNRS* were markedly upregulated, and those of *psaA*, *psbA*, and *ycf* were significantly increased. In contrast, most of the remaining chloroplast development-related genes showed no significant differences in expression between NIL-*qCD2* and Liaojing454.

Among the genes involved in chlorophyll biosynthesis and photosynthesis, the transcript levels of *CAO*, *CHLI*, *CHLH*, *RpoA*, *OsGK*, and *OsPRR3* were significantly upregulated in NIL-*qCD2*. Most of the remaining chlorophyll biosynthesis- and photosynthesis-related genes exhibited no significant differences in expression between the two genotypes (Figure 8B).

Among the leaf senescence-related genes examined, the transcript levels of *OsCATB*, *Os03g15840*, *LOX2*, *AOS2*, *PR1b*, and *Lhcb1* were markedly increased in NIL-*qCD2*, and those of *Pr1a*, *Lhcb4*, and *GAPDH* were also significantly upregulated (Figure 8C). Collectively, these results indicate widespread transcriptional alterations in genes associated with chloroplast development, chlorophyll metabolism, photosynthesis, and leaf senescence in NIL-*qCD2*.

## 4. Discussion

Rice grain yield is largely determined by photosynthetic productivity during the reproductive stage, in which chlorophyll plays a central role in light harvesting and carbon assimilation [3,5]. Premature chlorophyll degradation accelerates leaf senescence, shortens the effective photosynthetic period, and ultimately limits dry matter accumulation and grain filling, leading to reductions in both grain yield and grain quality [41,42,43,44,45]. Consistent with these observations, NIL-*qCD2* exhibited accelerated chlorophyll loss after heading, accompanied by pronounced alterations in chloroplast ultrastructure, reduced chlorophyll fluorescence parameters, and significant decreases in yield- and quality-related traits. These results indicate that the physiological effects associated with *qCD2* extend beyond chlorophyll metabolism and are closely related to the maintenance of photosynthetic capacity during grain filling.

The altered RVA profile, increased chalkiness, and reduced grain dimensions in NIL-*qCD2* suggest that premature senescence may have impaired assimilate supply and endosperm starch deposition during grain filling. However, starch composition and starch-biosynthetic enzyme activities were not measured. Therefore, the RVA changes should be interpreted primarily as a possible indirect source–sink consequence of premature senescence rather than evidence that *qCD2* directly regulates starch metabolism.

Leaf senescence is a genetically programmed developmental process that coordinates nutrient remobilization from senescing leaves to developing grains. However, premature senescence disrupts this balance by accelerating chloroplast degradation, chlorophyll catabolism, and photosynthetic decline, thereby reducing assimilate supply for grain development [6,7,15]. Increasing evidence has demonstrated that leaf senescence is regulated through interconnected pathways involving chloroplast development, chlorophyll metabolism, phytohormone signaling, ROS homeostasis, epigenetic modification, and transcriptional regulation [46,47,48,49]. Recent functional studies have identified several transcriptional modules involved in these processes. *OsNAC103* positively regulates both natural and dark-induced leaf senescence, whereas *ONAC016* promotes chlorophyll degradation through the ABA-responsive *OsNAP* pathway. In addition, disruption of *OsDERF8* delays senescence, alleviates chloroplast damage, and increases grain yield through altered jasmonate signaling. Epigenetic regulation is also involved, as melatonin-deficient rice mutants exhibit premature senescence during grain filling, together with DNA methylation changes and transcriptional reprogramming of chlorophyll metabolism, carbon metabolism, and redox-related genes [50,51,52,53,54]. Li et al. [17] further demonstrated that mutation of *OsAGO2* accelerates reproductive-stage leaf senescence through activation of the Os*NAC300*–*OsNAP* regulatory pathway. Consistent with the chloroplast- and photosynthesis-associated phenotypes described in these senescence mutants, NIL-*qCD2* exhibited accelerated chlorophyll loss, disrupted chloroplast ultrastructure, and reduced photosynthetic performance. Although the causal gene underlying *qCD2* has not yet been identified, these observations suggest that *qCD2* may participate in regulatory networks associated with chloroplast maintenance and leaf senescence. Further functional analyses will be required to determine the molecular mechanism responsible for the *qCD2*-associated phenotype.

Chlorophyll content, a key physiological trait closely associated with photosynthetic capacity, is quantitatively controlled by multiple genes and strongly influenced by environmental conditions. Previous studies using diverse genetic populations under different environmental conditions have identified numerous QTLs associated with chlorophyll content and stay-green traits across all 12 rice chromosomes, substantially improving our understanding of the genetic architecture underlying chlorophyll metabolism and leaf senescence [22,27,28,46]. Notably, several QTLs have been reported on the short arm of chromosome 2. For example, four QTLs for stay-green traits were mapped to the interval between RM145 and RM322 using doubled-haploid lines derived from a cross between ‘Zhenshan 97’ and ‘Wuyujing 2’ [8]. Jiang et al. [23] identified *qCHM2*, a QTL controlling chlorophyll content from heading to maturity, between RM145 and RM29. In addition, *qCC2* was mapped to an approximately 1.4 Mb interval between RM3390 and RM7288 using progeny derived from an interspecific cross between *O. sativa* and *O. grandiglumis*. This QTL was consistently detected in the F_3_ and F_4_ generations at different developmental stages and was further confirmed by substitution mapping [24]. In the present study, *qCD2* was initially detected on the short arm of chromosome 2 in an F_2_ population derived from a cross between *Shennong0530-9* and Habataki and was subsequently validated and fine-mapped using RHL-derived populations from the same cross. Although *qCD2* was located in the same chromosome region as several previously reported QTLs for chlorophyll content and stay-green traits, its fine-mapped interval near RM3732 was physically distinct from the RM145–RM322, RM145–RM29, and RM3390–RM7288 intervals. These results suggest that *qCD2* may represent a distinct locus involved in the regulation of chlorophyll content. Ultimately, *qCD2* was delimited to a 54.0 kb genomic interval containing nine predicted genes, providing a solid foundation for candidate gene identification and subsequent functional validation. Based primarily on their functional annotations and potential biological relevance, several genes were considered as candidates underlying *qCD2*.

Among the nine predicted genes within the 54.0 kb interval, *LOC_Os02g02860* is one of the most plausible candidates because it encodes a putative glutamyl-tRNA synthetase. In plastids, glutamyl-tRNA is required for both protein translation and tetrapyrrole biosynthesis, which produces chlorophyll and heme. Therefore, allelic variation in *LOC_Os02g02860* may affect chlorophyll biosynthesis, chloroplast development, and photosynthetic capacity, consistent with the accelerated chlorophyll loss and abnormal chloroplast ultrastructure observed in NIL-*qCD2*. *LOC_Os02g02870* and *LOC_Os02g02880* may be associated with the temperature sensitivity of the *qCD2* phenotype. *LOC_Os02g02870* encodes the cold-shock-domain protein OsCSP1, which may participate in the regulation of RNA stability and translation in response to temperature changes, whereas *LOC_Os02g02880* is predicted to encode a FIONA1 homolog associated with mRNA m^6^A modification and may therefore affect the post-transcriptional regulation of chloroplast maintenance, stress responses, or leaf senescence. However, the function of *LOC_Os02g02880* in rice remains to be experimentally confirmed. The remaining genes may participate in ubiquitination, small-GTPase signaling, one-carbon metabolism, protein folding, or other currently unknown biological processes. Although their direct involvement in chlorophyll metabolism and leaf senescence has not been demonstrated, they cannot be excluded solely on the basis of functional annotation. Overall, *LOC_Os02g02860*, *LOC_Os02g02870*, and *LOC_Os02g02880* can be prioritized for further investigation.

An interesting finding of this study is that the chlorophyll-deficient phenotype associated with *qCD2* became progressively more severe under elevated temperature conditions. Previous studies have shown that high temperature accelerates leaf senescence by promoting chlorophyll catabolism, destabilizing chloroplast membranes, impairing photosystem activity, and enhancing ROS accumulation [47,48,49]. Our observations are consistent with these reports and suggest that the phenotypic effect associated with *qCD2* is temperature-sensitive. However, whether *qCD2* directly participates in temperature-responsive regulatory pathways remains unclear because the causal gene has not yet been identified. Future studies integrating candidate gene sequencing, expression profiling under different temperature conditions, gene editing, and genetic complementation will be essential for elucidating the molecular basis of the temperature-sensitive phenotype.

Although *qCD2* was detected in both the F_2_ and F_2_:_3_ populations, its PVE decreased from 14.53% to 5.90%. This difference may reflect variations in population size, environmental conditions, and phenotyping strategy. In the F_2_ population, SPAD values and genotypes were obtained from the same individuals, whereas F_2_:_3_ phenotypes were calculated as family means but assigned according to the corresponding F_2_ parental genotypes. Because progeny derived from heterozygous F_2_ plants continued to segregate, the family mean may not precisely represent a single genotype, thereby weakening genotype–phenotype correspondence and reducing the estimated QTL effect. Nevertheless, *qCD2* was repeatedly detected in the same genomic region with a consistent allelic effect, supporting its reproducibility.

Collectively, this study identified and fine-mapped a stable major QTL, *qCD2*, associated with chlorophyll degradation during the reproductive stage in rice. Physiological, cytological, and agronomic analyses consistently showed that allelic variation at *qCD2* was associated with differences in chlorophyll retention, chloroplast integrity, photosynthetic performance, grain yield, and grain quality. However, the *Shennong0530-9* allele characterized in this study was associated with undesirable premature senescence. Further map-based cloning, functional validation, and allelic diversity analysis will be required to identify favorable *qCD2* allele(s) and develop tightly linked or functional markers. Thus, the present findings primarily provide a genetic basis and valuable materials for causal-gene identification, and may facilitate the prospective development of marker-assisted strategies for improving leaf longevity, photosynthetic performance, and grain productivity in rice.

## 5. Conclusions

In this study, we identified *qCD2* as a stable major quantitative trait locus controlling chlorophyll content and degradation during the reproductive stage in rice. Fine mapping delimited *qCD2* to a 54.0 kb genomic interval containing nine predicted genes. Physiological and cytological analyses showed that the *qCD2* locus was associated with accelerated chlorophyll loss, disrupted chloroplast ultrastructure, reduced photosynthetic performance, and concomitant decreases in grain yield and quality. Moreover, the *qCD2*-associated phenotype was markedly enhanced under elevated-temperature conditions, indicating that the effect of *qCD2* is temperature-dependent.

Collectively, these findings provide new insights into the genetic regulation of chlorophyll maintenance and degradation during reproductive development in rice and establish a foundation for identifying the causal gene underlying *qCD2*. The genetic materials developed in this study will facilitate functional characterization of *qCD2* and may support marker-assisted breeding of rice cultivars with improved photosynthetic performance, delayed leaf senescence, stable yield, and enhanced grain quality.

## Figures and Tables

**Table 1:** Markers used for the fine mapping of *qCD2*.

Marker	Forward Primer (5′–3′)	Reverse Primer (5′–3′)	Physical Position (bp)
RM2770	TAGGCCCTGATTAGTTTCC	ATATATGTGTCCCTTCTCCATAC	805,884
RM12353	TACTTCTCCCACTTGGACTTTGC	GATCAGTTCTTGAGATGGGATGG	1,078,287
ID10	ACTAGCATGTGTCATATCCAGTAC	GGGAACCAGTGAAAAGTCA	1,089,296
RM0173	TCATCTTTTTCATCTCGGGG	ATCTGGGTCACATGGAGAGC	1,103,244
RM6367	GCAACCACGACATCAAAGAAACC	GGAGGTTAGTGCTTCGGAGTGG	1,132,284
RM12363	TGCCAGGATGGACAATTAGATGC	CTCTGAGATTGAGGCTGCTCTGG	1,199,026
RM233A	CCAAATGAACCTACATGTTG	GCATTGCAGACAGCTATTGA	2,069,785
MM0269	ACTGGTGTTTACTCCGTGGG	GGTCTCCAAATCCAGCAGTG	2,111,935

**Table 2:** Phenotypic analysis of CD values in the parental, F_2_, and F_2_:_3_ populations.

Traits	Populations	Parents		F_2_ and F_2:3_ Populations			
		Habataki	Shennong 0530-9	Mean ± Standard Deviation	Variation	Skewness	Kurtosis
Degradation Index	F_2_	0.90	0.87	0.84 ± 0.14	0.36–1.11	−0.59	0.13
CD	F_2:3_	0.95	0.86	0.76 ± 0.085	0.51–0.95	−1.11	1.86

**Table 3:** QTL Analysis of Chlorophyll Content at Different Growth Stages.

Stages	Chr	Marker	LOD Value ^a^		PVE (%) ^b^		Add Effect ^c^		Dom ^d^	
			F_2_	F_2:3_	F_2_	F_2:3_	F_2_	F_2:3_	F_2_	F_2:3_
Chlorophyll content of tiller stage	*qCT2*	STS6-R2M50	2.51		10.77		1.12		0.73	
	*qCT3*	R3M10-R3M23	4.60		11.66		−1.44		−0.11	
	*qCT7*	RM427-RM8263	2.79		7.69		0.29		1.69	
	*qCT10*	R10M40-RM3451	3.14		6.61		−0.82		−0.73	
	*qCJ1*	RM1061-RM7180		2.89		15.15		−3.69		2.98
Chlorophyll content of joint stage	*qCJ2a*	STS5-RM1347	4.05	4.23	10.92	12.00	−1.80	−1.51	1.94	1.54
	*qCJ2b*	STS6-R2M50		3.26		14.20		1.24		1.08
	*qCJ3*	R3M10-R3M23	4.31	2.46	12.12	8.81	−1.56	−1.42	0.89	−0.35
	*qCJ6*	RM587-RM6836	2.74		12.15		1.79		1.21	
	*qCJ9*	STS13-RM9	2.95		7.61		−1.20		1.38	
	*qCJ10*	R10M40-RM3451		3.15		9.49		−1.08		−0.70
	*qCJ12*	R12M27-RM3739	2.42		6.99		−0.74		−1.82	
Chlorophyll content of heading stage	*qCH2a*	STS5-RM1347	2.69	2.17	12.46	10.75	−1.39	−1.46	0.35	3.11
	*qCH2b*	STS6-R2M50	2.96	8.33	8.39	15.85	0.39	−0.42	2.31	12.24
	*qCH3a*	R3M10-R3M23	7.80		24.65		−2.66		0.97	
	*qCH3b*	RM2334-RM5172	2.63		5.83		0.51		−2.02	
	*qCH4*	STS9-RM1359		2.09		6.82		−1.80		1.73
	*qCH5*	R5M13-RM430		7.38		17.71		6.23		6.82
	*qCH10*	R10M40-RM4771	2.81	5.35	11.97	29.14	−1.32	4.13	−3.66	2.08
Chlorophyll content of maturing	*qCM2*	STS5-RM1347	2.41	3.81	12.65	10.11	−3.02	−2.84	1.41	−1.01
	*qCM3a*	R3M10-R3M23	2.80	2.51	5.65	24.48	−2.28	−0.65	−1.08	−6.18
	*qCM3b*	RM2334-RM5172	2.68		5.16		−0.48		−3.05	
	*qCM6*	RM587-RM6836	4.51		23.04		0.80		6.61	
	*qCM8*	RM6208-RM331	2.49	2.13	4.51	10.50	−1.95	1.15	0.01	−4.28
	*qCM9*	RM9-STS14	2.43		8.18		−0.31		−4.04	
Chlorophyll degradation	*qCD1*	RM6902-RM583		3.80		49.08		0.26		0.37
	*qCD2*	STS-RM3732	2.60	2.09	14.53	5.90	0.14	0.05	0.24	0.05
	*qCD3*	R3M23-RM2334		2.68		17.40		0.09		0.14
	*qCD5*	R5M13-RM430		6.06		47.76		0.14		0.54
	*qCD6*	RM6836-RM7193		2.66		9.76		0.05		−0.11
	*qCD8*	RM6208- RM331	3.79	2.03	7.52	9.47	−0.05	0.08	−0.02	0.09

^a^. Maximum LOD score for each QTL; ^b^. Percentage of phenotypic variance explained by each QTL; ^c^. Additive effect estimated by QTL IciMapping. Habatake was designated as P1 and *Shennong0530-9* as P2; therefore, a positive value indicates that the Habatake allele increases the trait value, whereas a negative value indicates that the Shennong0530-9 allele increases the trait value; ^d^. Dominance effect estimated by QTL IciMapping. A positive value indicates that the heterozygous genotypic value is higher than the mean of the two homozygous genotypic values, whereas a negative value indicates that it is lower.

**Table 4:** Gene annotation of nine open reading frames within the 54.0 kb region in the fine-mapped region on chromosome 2.

ORF	Genes ID	Gene Function Annotation	DNA (cDNA) Length	Protein Length (aa)	Protein Weight	Location
ORF1	*LOC_Os02g02830*	Ubiquitin-conjugating enzyme, putative, expressed	1178 (447)	149	16,600.2	1,078,475–1,079,652
ORF2	*LOC_Os02g02840*	Ras-related protein, putative, expressed	3771 (594)	198	21,624.0	1,084,283–1,080,513
ORF3	*LOC_Os02g02850*	Bifunctional protein fold, putative, expressed	3469 (1116)	372	39,358.8	1,089,626–1,086,158
ORF4	*LOC_Os02g02860*	Glutamyl-tRNA synthetase, putative, expressed	5682 (1704)	568	62,863.8	1,095,993–1,090,312
ORF5	*LOC_Os02g02870*	Glycine-rich protein 2, putative, expressed	1342 (726)	242	22,723.4	1,104,306–1,102,965
ORF6	*LOC_Os02g02880*	Expressed protein	5758 (1401)	467	50,743.6	1,111,999–1,106,242
ORF7	*LOC_Os02g02890*	Peptidyl-prolyl cis-trans isomerase, putative, expressed	1078 (519)	173	18,361.0	1,116,059–1,117,136
ORF8	*LOC_Os02g02900*	Expressed protein	1893 (798)	266	29,333.9	1,123,522–1,125,414
ORF9	*LOC_Os02g02910*	Expressed protein	6596 (1956)	652	71,641.0	1,132,613–1,126,021

**Table 5:** Comparison of yield and grain quality traits between Liaojing454 and NIL-qCD2.

Traits	Liaojing454	NIL-*qCD2*	Traits	Liaojing454	NIL-*qCD2*
Biological yield (g)	49.66 ± 3.62	46.52 ± 2.50 **	Brown rice rate (%)	80.78 ± 0.22	80.06 ± 0.08
Grain yield (g)	22.65 ± 1.30	20.36 ± 1.58 *	Milled rice rate (%)	74.11 ± 0.57	71.51 ± 0.27 *
Plant height (cm)	108.5 ± 5.40	109.25 ± 6.20	Chalky grain rate (%)	15.05 ± 5.72	19.01 ± 0.14 **
No. of effective tillers per plant	13.87 ± 0.83	12.75 ± 0.70	Chalkiness (%)	8.30 ± 3.39	14.20 ± 0.00 **
Panicle length (cm)	18.58 ± 0.19	17.20 ± 0.66	Whiteness	27.30 ± 2.26	16.60 ± 1.56 **
Grain number per panicle	172.03 ± 1.23	161.43 ± 4.25 *	Peak viscosity (cP)	2833.50 ± 0.71	2548.50 ± 33.23 *
Seed setting rate (%)	95.62 ± 5.66	93.25 ± 4.51	Hot paste viscosity (cP)	2125.50 ± 7.78	1813.00 ± 33.94 *
1000-grain weight (g)	24.41 ± 0.34	23.25 ± 0.27 *	Breakdown viscosity (cP)	708.00 ± 7.07	735.50 ± 67.17 *
Grain length (mm)	7.11 ± 0.25	7.06 ± 0.16	Setback viscosity (cP)	1136.50 ± 10.61	1284.50 ± 45.96 *
Grain width (mm)	3.25 ± 0.15	3.04 ± 0.17 *	Peak time (min)	6.30 ± 0.04	6.13 ± 0.00 *
Grain thickness (mm)	2.18 ± 0.69	2.02 ± 0.01 *	Pasting temperature (°C)	83.28 ± 0.04	86.15 ± 0.56 *

Note: Data are presented as the mean ± SD of three independent field plots (*n* = 3). The mean of five plants sampled from each plot was used as one biological replicate. * and ** indicate significant differences at *p* < 0.05 and *p* < 0.01, respectively, according to Student’s *t*-test.

**Table 6:** Characterization of photosynthetic and chlorophyll fluorescence kinetic parameters of Liaojing454 and NIL-qCD2.

Traits	Pn (μmolCO_2_m^−2^s^−1^)	Gs (molH_2_Om^−2^s^−1^)	Ci (μmolCO_2_m^−1^)	Tr (mmolH_2_Om^−2^s^−1^)	F0	Fv/FM	qN	ETR
Liaojing454	21.38 ± 1.64 aA	1.12 ± 0.18 aA	301.83 ± 1.30 aA	8.80 ± 0.37 aA	177.67 ± 5.39 aA	0.84 ± 0.01 aA	0.55 ± 0.16 aA	243.73 ± 17.59 aA
NIL-*qCD2* green	19.59 ± 1.79 aA	0.67 ± 0.21 bA	286.23 ± 8.49 aA	7.44 ± 0.30 bA	180.23 ± 4.14 aA	0.84 ± 0.01 aA	0.49 ± 0.12 aA	231.62 ± 20.83 aA
NIL-*qCD2* yellow	14.72 ± 0.35 bB	0.55 ± 0.31 bB	287.74 ± 20.28 aA	6.68 ± 1.59 bA	194.22 ± 9.19 bB	0.83 ± 0.01 aA	0.30 ± 0.08 bB	153.48 ± 39.87 bB

Note: Data are presented as mean ± standard deviation. One-way analysis of variance (ANOVA) was performed, followed by Duncan’s multiple range test. Within the same column, values followed by different lowercase letters are significantly different at *p* < 0.05, whereas values followed by different uppercase letters are significantly different at *p* < 0.01. Values followed by the same lowercase or uppercase letters are not significantly different at the corresponding significance level.

## Data Availability

The original contributions presented in the study are included in the article, and further inquiries can be directed to the corresponding authors.

## Supplementary Materials

The following supporting information can be downloaded at: https://www.mdpi.com/article/10.3390/biology15161384/s1, Table S1. RT-qPCR primer sequences for chlorophyll metabolism, photosynthesis-, and leaf senescence-related genes in rice.
