# Supplementary material for: Identification and Fine Mapping of qCD2, a Major QTL Governing Leaf Premature Senescence in Rice (Oryza sativa L.)
Source: Biology (Basel). 2026 Aug 13;15(16):1384. doi: 10.3390/biology15161384 (PMC13510241; doi:10.3390/biology15161384)
Supplement: Supplementary file 1 [file biology-15-01384-s001.zip › biology-4463623-supplementary.pdf]

Table S1. RT-qPCR primer sequences for chlorophyll metabolism, photosynthesis-, and leaf senescence-related genes in rice.

Target gene	Gene number	Forward primer（5′ –3′）	Reverse primer（5′–3′）	Source
psaA	OrsajCp025	GCGAGCAAATAAAACACCTTTC	GTACCAGCTTAACGTGGGGAG	Dong et al., 2013
psbA	OrsajCp002	CCCTCATTAGCAGATTCGTTT	ATGATTGTATTCCAGGCAGAGC	Dong et al., 2013
rbcL	OrsajCp023	CTTGGCAGCATTCCGAGTAA	ACAACGGGCTCGATGTGATA	Dong et al., 2013
ycf	LOC_Os06g39744	GTGGAATGTAGGCAGTGGTTATGAC	CGCATAAGGAATCGAAGAAAGAC	This study
psaB	OrsajCp024	CGAACCTATATTTGCTCAATGGATAC	GATGAACCAAGAAATCTCCAGGTC	This study
V1	Os03g0656900	TGGAGGTCGGGACAGAGGA	CGAGGAGCACCACCATCAC	Dong et al., 2013
SPP	Os06g0625400	CGGAGAGGAAACATAATGAC	ATAGGCATTTGTCTTTGTCTC	This study
OsPPR	Os09g0413300	CTAAGACCGAATGACAAATGC	GCACTGCCAACAAGAATACC	This study
TSL11	Os12g0563200	CCAAACCTTGTCGATCCCTTC	CTCGAAGACGATGGCTTCTTCT	This study
OsPOLP	Os08g0175300	ACCGGTGCTTTCAGGCTTGG	GCTGACTGATAATCACACG	This study
RpoTp	Os06g0652000	TCCTCTATGTCGAGCAAGGAT	GAAAGAATGTCTGGACTTTG	Dong et al., 2013
RNRS	Os06g0257450	GCCAAAATCCATTCCAATTC	GGAGATGTACACGAGGAGATTG	This study
osbel	Os06g0108900	GGGAAGGGCAAGAACCAGAT	CCTCATCGACGTATTGATTG	This study
HEMA1	Os10g0502400	CGCTATTTCTGATGCTATGGGT	TCTTGGGTGATGATTGTTTGG	Wu et al., 2007
CHLD	Os03g0811100	GCTTGCAGAAAAGCTACACAAGC	AGGCCGTGAGCTAAAGGAGC	This study
YGL	Os05g0349700	ATGAACCTTACCGTCCTATTCCTT	GGACCCACCAACAGCAAGAT	This study
PORA	Os04g0678700	TGTACTGGAGCTGGAACAACAA	GAGCACACGCAAAATCCTAGACG	Wu et al., 2007
CAO	Os10g0567400	GATCCATACCCCGATCGACAT	CGAGAGACATCCGGTAGAGC	Wu et al., 2007
CHLI	Os03g0563300	AGTAACCTTGGTGCTGTG	AATCCATCAACATTCAACTCTG	This study
CHLH	Os03g0323200	CTATACATTCGCCACACT	TATCACACAACCTCCCAAG	This study
OsDVR	Os03g0351200	CGAGCCCAGGTTCATCAAGGTGC	CCTCCCGATCTTGCCGAACTCC	This study
OsGK	Os03g0320900	CGACAAGCAGAGCGAAGCG	AGGTTGCTGCTCCTTGAATGT	Dong et al., 2013
cab1R	Os09g0346500	AGATGGGTTTAGTGCGACGAG	TTTGGGATCGAGGGAGTATTT	Wu et al., 2007
cab2R	Os03g0592500	TGTTCTCCATGTTCGGCTTCT	GCTACGGTCCCCACTTCACT	Wu et al., 2007
rbcS	Os12g0274700	TCCGCTGAGTTTTGGCTATTT	GGACTTGAGCCCTGGAAGG	Wu et al., 2007
OsPPR3	Os01g0559500	TCCTCATGTGTCCCAGGATCCAAT	AGCTCTTGTGGAATCTCACCCTGA	This study
RpoA	OrsajCp059	GCGTCTTTATTATGGTCGTTTCA	CTCCCAAATTTTGCATGTGTGAT	Dong et al., 2013
OsCATA	Os02g0115700	GCCGGATAGACAGGAGAGGT	TCTTCACATGCTTGGCTTCA	This study
OsCATB	Os06g0727200	GGTGGGTTGATGCTCTCTCA	ATTCCTCCTGGCCGATCTAC	This study
LOC_Os03g15840	LOC_Os03g15840	TGGACCCACATGGAGTCTTA	AACACCTTTGTTCCAAAGCC	This study
OsPR1a	Os12g0555500	GGTGTGGGAAGCACATACAA	TCCGTCGAGTGTGACTTGAG	This study
LOX2	Os08g0508800	AGGGCACGCCTAGCAAAC	CTTGTCGATGACGAAGAAGGACGGG	This study
AOS2	Os12g0178100	GCTACTATAGAGCATATTATG	CTAATGGAGAGCACAACTCA	This study
Pr1a	Os07g0129200	TATGCTATGCTACGTGTTTATGC	CACTAAGCAAATACGGCTGACA	This study
PR1b	Os01g0382000	GGCAACTTCGTCGGACAGA	CCGTGGACCTGTTTACATTTTCA	This study
PR5	Os03g0663500	ACGACCAGACGAGCACCTTCA	TCGATCAGATGCCAGCTAACAAG	This study

Lhcb1	Os01g0600900	CCATGTTCTCCATGTTCGGCTTCT	TAGGCCCAGGCGTTGTTGTTGA	This study
Lhcb4	Os07g0558400	TACCTGCAGTTCGAGCTGGAC	AGGCCGAACACCTCGGTGTA	This study
GAPDH	Os04g0486600	AAGCCAGCATCCTATGATCAGATT	CGTAACCCAGAATACCCTTGAGTTT	This study
Actin	Os03g0718100	AGGAAGGCTGGAAGAGGACC	CGGGAAATTGTGAGGGACAT	Wu et al., 2007

Table note: Gene-specific primers used for quantitative real-time PCR. Primer sequences are shown in the 5′–3′ orientation. *Actin* was used as the internal reference gene for normalization of relative transcript levels.Primer pairs labeled “This study” were newly designed in the present study..
